# Movement Behavior during Pregnancy and Adverse Maternal–Fetal Outcomes in Women with Gestational Diabetes: A Pilot Case-Control Study

**DOI:** 10.3390/ijerph18031114

**Published:** 2021-01-27

**Authors:** Sávio F. Camargo, Juliana D. Camargo, Daniel Schwade, Raíssa M. Silva, Maria da Conceição M. Cornetta, Ricardo N. Cobucci, Eduardo C. Costa

**Affiliations:** 1 Graduate Program in Health Sciences, Federal University of Rio Grande do Norte, Natal 59010-000, Brazil; saviocamargo@gmail.com; 2 Maternity Hospital-School Januário Cicco, Federal University of Rio Grande do Norte, Natal 59010-000, Brazil; juliana_ily@hotmail.com (J.D.C.); mcornetta@hotmail.com (M.d.C.M.C.); drcobucci@ufrn.edu.br (R.N.C.); 3 Faculty of Kinesiology and Recreation Management, Health, Leisure, and Human Performance Research Institute, University of Manitoba, Winnipeg, MB R3B 2E9, Canada; araujod@myumanitoba.ca; 4 Department of Physical Education, Federal University of Rio Grande do Norte, Natal 59010-000, Brazil; raissamelonutricionista@gmail.com; 5 Graduate Program in Biotechnology, Potiguar University, Natal 59010-000, Brazil

**Keywords:** gestational diabetes, health outcomes, physical activity, sedentary behavior

## Abstract

Gestational diabetes mellitus (GDM) is a major complication in pregnancy. GDM is associated with a higher risk for adverse maternal–fetal outcomes. Associations between movement behavior, including physical activity (PA) and sedentary behavior (SB), and maternal–fetal outcomes are still unclear. The objective of this study was to investigate associations between movement behavior and adverse maternal–fetal outcomes in women with GDM. A total of 68 women with GDM (20–35 weeks, 32.1 ± 5.8 years) were included in this pilot case-control study. The cases were defined by the presence of an adverse composite maternal–fetal outcome (preterm birth, newborn large for gestational age, and neonatal hypoglycemia). Controls were defined as no adverse maternal–fetal outcome. PA intensities and domains, steps/day (pedometer), and SB were analyzed. A total of 35.3% of participants showed adverse maternal–fetal outcomes (n = 24). The controls showed a higher moderate-intensity PA level than the cases (7.5, 95%CI 3.6–22.9 vs. 3.1, 95%CI 0.4–10.3 MET-h/week; *p* = 0.04). The moderate-intensity PA level was associated with a lower risk for adverse maternal–fetal outcomes (OR 0.21, 95%CI 0.05–0.91). No significant associations were observed for other PA and SB measures (*p* > 0.05). In conclusion, moderate-intensity PA during pregnancy seems to have a protective role against adverse maternal–fetal outcomes in women with GDM.

## 1. Introduction

Gestational diabetes mellitus (GDM) is the most common complication in pregnancy. It is a risk factor for adverse maternal–fetal outcomes such as preterm birth, newborn large for gestational age (LGA), and neonatal hypoglycemia [1,2,3]. Previous studies have shown that physically active women before [4,5] and during pregnancy [6,7] have lower weight gain and risk of adverse maternal–fetal outcomes than their physically inactive peers. The clinical guidelines recommend that pregnant women, including those with GDM, perform 150 min/week of moderate–vigorous physical activity (MVPA) [8]. A recent meta-analysis conducted by Brown, Ceysens, and Boulvain [9], including 11 randomized controlled trials (RCTs), concluded that exercise interventions were associated with both reduced fasting and postprandial blood glucose concentrations compared to control interventions. However, the authors state that the current evidence is unclear due to the wide variety of exercise interventions, making it difficult to identify evidence of sufficiently high quality to be able to determine differences between the exercise and control groups for health outcomes in women with GDM.

Increasing physical activity (PA) levels may reduce the impact of poor glycemic control and excessive weight gain on adverse outcomes associated with GDM. Regular PA may reduce the maternal–fetal consequences associated with maternal hyperglycemia such as hypertension and macrosomia. Thus, PA benefits seem to occur not only for pregnant women who receive treatment, but also for subsequent generations [10,11]. Recent studies have investigated the influence of different aspects of movement behavior (PA and sedentary behavior) on maternal and child outcomes in GDM [12,13]. Sedentary behavior (SB) is defined as activities performed in a sitting, reclining, or lying posture with 1.5 or less metabolic equivalents (METs). SB is an important risk factor for GDM, while MVPA plays a protective role [14]. Previous studies have shown that higher PA and lower SB are associated with a reduced risk of adverse maternal–fetal outcomes [11,15,16]. However, further research is still needed to determine the benefits of the different movement behavior components on maternal–fetal outcomes in women with GDM. Therefore, this pilot study aimed to investigate the associations between PA (domains and intensities) and SB measures during pregnancy and the risk of adverse maternal–fetal outcomes in women with GDM.

## 2. Materials and Methods

### 2.1. Study Design

This is a pilot case-control study reported in accordance with the Strengthening the Reporting of Observational Studies in Epidemiology (STROBE) [17] statement. This study was conducted between June 2018 and December 2019 at the Maternity School Januário Cicco, Federal University of Rio Grande do Norte, Natal, RN, Brazil. This study was approved by the Research Ethics Board at the Onofre Lopes University Hospital (protocol 66795417.6.0000.5292) and conducted according to the Declaration of Helsinki. The participants were informed about all study procedures and provided written informed consent.

### 2.2. Participants

Data from delivery registration books of obstetric centers from the Januário Cicco Maternity School, Dr. José Pedro Bezerra Hospital, and the Dr. Araken Irerê Pinto Municipal Maternity of 68 women with GDM were obtained from October 2018 to December 2019 in order to assess the delivery mode (i.e., vaginal or cesarean) and the occurrence of preterm birth, newborn LGA, and neonatal hypoglycemia. The cases were defined as at least one of the above-mentioned adverse maternal–fetal outcomes (i.e., preterm birth, newborn LGA, or neonatal hypoglycemia). The controls were defined as no adverse maternal–fetal outcome. These 68 participants were included in this pilot case-control study because they had participated in a previous cross-sectional study in which a detailed PA and SB assessment was conducted during their pregnancy (20–35 weeks of gestational age). All participants had been recruited in their first medical appointment in high-risk prenatal care at the Januário Cicco Maternity School, Federal University of Rio Grande do Norte, Natal, RN, Brazil. The inclusion criteria were (i) being diagnosed with GDM according to the 2018 World Health Organization diagnostic criteria [18] and (ii) single gestation between 20 and 35 weeks. The exclusion criteria were (i) previous history of type 1 or type 2 diabetes and (ii) urinary tract infection in prenatal care.

### 2.3. Pregnancy Data

The information related to the pregnancy period of all participants was retrieved from medical records and face-to-face interviews which were performed with the participants between their 20 and 35 weeks of gestational age as part of a previous cross-sectional study. The following variables were considered for the present study: age, educational level, living arrangement with partner, family income, parity, previous preterm birth, previous GDM, maternal family history of diabetes, self-reported pregestational body mass index (BMI), maternal weight gain, gestational age, insulin therapy, hypertension diagnosis, number of prenatal visits, and dietary intake. All participants were assessed in a face-to-face interview in order to identify their usual dietary intake. A validated Brazilian food frequency questionnaire [19] including 13 different food groups (vegetables/legumes, fresh fruits/fruit juices, chestnuts/nuts/oilseeds, olive oil/vegetable oils, whole grains/cereals, sausage/canned meat/preserved meat/processed meats, milk/dairy products, fish/omega-3 sources, red meat with apparent fat, soft drinks/artificial beverages, ice cream/sweet cookies/stuffed cookies, chips/breaded pies/fried snacks, and other ultraprocessed foods) was used to assess the participants’ dietary intake during a usual week period (see covariates in Table S1). The participants’ dietary intake considering the 13 different food groups was classified as “appropriate” or “inappropriate” according to the dietary guidelines for the Brazilian population [20].

Similar to the above-mentioned variables, PA and SB measures were obtained during the 20 to 35 weeks of gestational age of all participants. Energy expenditure (MET-h/week) from different PA intensities (light, moderate, and vigorous) and domains (household/caregiving, occupational, sports/exercise, and transportation) were obtained from the validated Portuguese version of the Pregnancy Physical Activity Questionnaire (PPAQ) [21,22]. The following PA intensities were considered: (i) light, from 1.5 to <3.0 METs; (ii) moderate, from 3.0 to 6.0 METs; and (iii) vigorous, >6.0 METs [21,22]. The following PA domains were additionally considered: (i) household/caregiving activities including housekeeping and taking care of children, older adults, or pets; (ii) occupational activities including PA performed during work; (iii) sports/exercise PA including exercise and activities performed for fun; and (iv) transportation PA including walking to or from somewhere [21,22]. The pedometer-measured PA level (steps/day) was considered in addition to energy expenditure from PA intensities and domains in the present study. Pedometers (Omron, HJ-321 Tri-Axis Alvita, USA) were individually adjusted for the participants based on their stride length, weight, and height according to the manufacturer’s instructions. The quantity of steps/day was assessed during a 1-week period for all participants. The SB was assessed by the Longitudinal Aging Study Amsterdam Sedentary Behavior Questionnaire (LASA-SBQ) [23].

### 2.4. Adverse Maternal–Fetal Outcomes

As previously mentioned, the presence of a composite adverse maternal–fetal outcome (including preterm birth and/or newborn LGA and/or neonatal hypoglycemia) was used as the criterion to define the cases. Preterm birth was defined as prior to 37 weeks of gestation according to the American College of Obstetricians and Gynecologists (ACOG) [24], large for gestational age implies a birth weight equal to or greater than the 90th percentile for a given gestational age according to ACOG [25], and neonatal hypoglycemia was defined as the occurrence of at least one report below 45 mg/dL during the first 24 h of life according to the Brazilian Pediatric Society [26].

### 2.5. Statistical Analysis

Data normality was tested using the Shapiro–Wilk test. Results were expressed as mean ± standard deviation (SD) for the parametric data and median and 25th to 75th percentiles for the nonparametric data. Categorical data were expressed as absolute and relative frequencies. Independent sample t-test or Mann–Whitney U test, chi-squared test, or Fisher’s exact test was used in the preliminary analysis. As an exploratory analysis, when the movement behavior measure (PA domains and intensities, steps/day, and SB) was different between cases and controls, unadjusted and adjusted odds ratios (ORs) and their respective confidence intervals (CIs) for the composite adverse maternal–fetal outcome were calculated by logistic regression analysis. Thus, the whole sample was divided into tertiles for each movement behavior measure for this analysis (tertile 1 as reference for comparisons). The *p* < 0.20 in the bivariate analysis was used as a criterion to include the variable as a confounding factor in the adjusted analysis. Parameter estimates were obtained using maximum likelihood techniques and their respective 95% CIs for the composite adverse maternal–fetal outcome. Statistical significance was set at 5%. Statistical procedures were performed using IBM SPSS Statistics for Windows, v.25.0 (IBM Corp., Armonk, NY, USA).

## 3. Results

A total of 10 out of 92 potentially eligible participants declined to participate in the study, while 10 were excluded due to not presenting a compatible gestational age, and 4 more were excluded due to urinary tract infection. Thus, 68 participants were included in the final analysis. Table 1 shows the characteristics of case and control participants. The participants from the case and control groups showed similar overall characteristics. Supplementary Table S1 shows the prevalence of appropriate dietary intake from different food sources of case and control participants. No significant differences were found between the case and control participants (*p* > 0.05). A total of 35.3% (n = 24) of participants showed adverse maternal–fetal outcomes. The occurrence of preterm birth was 13.2% (n = 9), newborn LGA was 11.8% (n = 8), and neonatal hypoglycemia was 17.6% (n = 12).

Table 2 shows the PA and SB measures of case and control participants. The case participants showed lower moderate-intensity PA levels than the control participants (*p* < 0.05). The additional PA (domains, intensities, and steps/day) and SB (min/day) measures were not different between the case and control participants (*p* > 0.05). Regarding the PA domains, 47, 3, 1, and 44 participants did not report leisure/exercise, transportation, household/domestic, and occupational activities, respectively. Regarding the PA intensities, all participants reported performing light PA, but 13 and 62 participants did not report performing moderate and vigorous PA, respectively. Five participants had technical issues with the pedometers and therefore their data were not included in the study.

Table 3 shows the OR for adverse maternal–fetal outcomes according to tertiles of energy expenditure in moderate-intensity PA (MET-h/week). Participants with the highest moderate-intensity PA level showed lower risk (OR = 0.21, 95%CI 0.05–0.91) for adverse maternal–fetal outcomes compared to participants with the lowest moderate-intensity PA level in the multivariate-adjusted analysis.

## 4. Discussion

This study investigated the associations between the measures of PA (intensities, domains, and step count) and SB during pregnancy and the risk for adverse maternal–fetal outcomes in women with GDM. The main finding was that the moderate-intensity PA level was negatively associated with the risk for adverse maternal–fetal outcomes.

Our study shows that the majority of women with GDM studied herein focused their activities on light intensity, followed by moderate PA and almost no vigorous PA. Based on previous data from the literature [27,28], the participants from our study had a low number of steps per day and high SB, with no differences between cases (those with adverse maternal–fetal outcomes) and controls (those without adverse maternal–fetal outcomes). The participants’ PA data are similar to those seen in studies carried out in developing countries [29] and are different from those seen in studies conducted in developed countries. For example, the energy expenditure data (MET-h/week) from the present study are much lower at light (67.8 vs. 90.3), moderate (5.8 vs. 48.3), and vigorous (0.0 vs. 1.4) intensities compared to a Canadian study [30]. Regarding vigorous PA, a study carried out in Vietnam showed that 3.0% of pregnant women performed PA at the above-mentioned intensity. However, only six women (8.8%) reported performing vigorous PA in our study. In the Avon Longitudinal Study of Parents and Children [31] in England, 48.8% practiced vigorous PA. The most common PA during pregnancy reported by these women was brisk walking, followed by swimming and antenatal exercise.

A large amount of the registered PA in the present study was performed in the domestic domain, including housekeeping and taking care of children, older adults, or pets, followed by activities in the transportation domain. It is common for most of the PA of pregnant women to be concentrated in the domestic domain regardless of the development level of the researched region, as shown in a study involving 2030 pregnant women developed in Vietnam [29] and in the Pregnancy, Infection, and Nutrition Study which investigated PA among 1482 pregnant women in the USA [32]. A modest portion of our sample registered leisure/exercise (28.0%) or occupational PA (35.3%). The values in the Pregnancy, Infection, and Nutrition Study were 60.7% and 30.2%, respectively. The low record of leisure/exercise PA may explain the lack of evidence of a difference between cases and controls in our study in contrast to several studies that point to leisure/exercise PA as a health protection factor in the pregnant population [33].

Observational studies investigating the association between PA and health outcomes in women with GDM are still scarce [34]. Higher volumes of moderate-intensity PA are associated with greater chances of achieving glycemic control [35,36]. It is known that MVPA results in greater glucose uptake by insulin-independent routes, and insulin sensitivity is also improved compared to light intensity activity, which results in improved glycemic control [37]. Pregnant women with well-controlled blood glucose tend to generate fetuses of appropriate weight for gestational age, as maternal hyperglycemia increases insulin secretion in the fetal pancreas. Insulin works as an anabolic hormone responsible for weight gain and neonatal hypoglycemia [38]. In addition, exercise during pregnancy can reduce the risk of excessive maternal weight gain [39]. The reduction of the mother’s body fat increases the transfer of oxygen and reduces the diffusion of carbon dioxide through the placenta, with a positive impact on fetal development [40]. This impact reduces the risk of LGA (without increasing the risk for small for gestational age) and the risk factors for adverse outcomes in GDM [36,41]. A recent umbrella review including 76 systematic reviews and meta-analyses on the benefits of PA during pregnancy points out strong evidence showing that moderate-intensity PA reduces the risk of excessive gestational weight gain [42], which supports the findings observed in the present study.

To date, light PA is less associated with potential benefits for the maternal–fetal binomial [43]. The main guidelines for PA delivered for pregnant women, such as the ACOG [44] and Canadian guidelines [45], recommend performing moderate-intensity PA. Our results did not register differences in light PA volume between cases and controls. Furthermore, we found no differences for vigorous PA, which can be explained by the almost total absence of records of vigorous PA in our sample. Physiological changes in pregnancy often make vigorous PA unviable [46]. In addition, clinicians usually recommend light aerobic activities for pregnant women such as light walking. Interestingly, there was no difference in the quantity of steps/day between cases and controls, which seems to suggest that the greater amount of moderate PA found in the controls is not necessarily related to the greater achievement of the most common type of PA during pregnancy, which is walking [31,40,47,48]. It is possible that the difference between the controls and the cases might be in the intensity and not in the volume of walking activities.

Our data also showed no differences in the PA domains. Recent studies claim that the current state of knowledge in the field is that prospective research is needed to establish the effects of specific types of exercise on maternal–fetal outcomes in women with GDM. Thus, effective methods of behavioral counseling on the ideal type, frequency, and intensity of exercises for women with different health conditions during pregnancy may be consolidated [47]. However, we can already consider that any type of PA with sufficient intensity and duration can have benefits for pregnant women with GDM [43,44]. Based on our findings in which moderate PA seems to protect against adverse maternal–fetal outcomes, it is clear that patients diagnosed with GDM need to be encouraged and instructed to be involved in PA in order to improve glycemic control and avoid excessive weight gain. After 22 weeks of an exercise program at moderate intensity three times a week in women with GDM, Barakat et al. [49] demonstrated that the exercise group had a 7% lower birth weight and a 12% lower maternal weight gain than the control group. The modest participation in moderate PA in the leisure/exercise domain in our study indicates that prenatal services for pregnant women with GDM should perform counseling/prescription focusing on this intensity and domain, as this is what the most solid evidence suggests [5,33,35,40,43,50] and the guidelines recommend [44,45]. Some activities which would fit into this category would be brisk walking, swimming, or weight training, given the precautions established for PA in diabetics [51] and during pregnancy [44,45]. Bo et al. [50] demonstrated that a program with individual oral/written recommendations to support a healthy diet and a brisk walking intervention of at least 20 min every day reduced maternal/neonatal adverse outcomes (perinatal and postpartum mothers, preterm delivery, newborn LGA, and any neonatal conditions that required an extended hospital stay) by 50% among women with GDM. Although GDM management advocated in prenatal services usually consists of diet and insulin therapy, PA must be included as a clinical approach. A PA intervention may act as a protective factor against adverse maternal–fetal outcomes and also help to reduce costs in the health care system, mainly by reducing the use of medications and hospitalizations.

Although this pilot study presents a more comprehensive analysis about the association between the movement behavior and adverse maternal–fetal outcomes in women with GDM, it has limitations that should be mentioned. The generalization of our findings should be interpreted with caution due to the inclusion of a sample recruited from only one reference center, which essentially serves the low-income population. No data were collected on impaired glucose tolerance during pregnancy, a variable that has a direct influence on adverse health outcomes in GDM. However, all participants were followed and treated during pregnancy from their first medical appointment in high-risk prenatal care. Those participants who did not maintain an appropriate glycemic control, including impaired glucose tolerance, were treated with insulin therapy (n = 25; 36.8% of the sample). It should be noted that the regression analysis in Table 3 includes insulin therapy as a confounding factor, which may reduce the uncertainty of our results regarding the absence of data of impaired glucose tolerance. The participants’ food intake was assessed as the usual food consumption, which does not enable a determination of energy intake, an aspect that may be associated with glycemic control. Finally, the movement behavior was only assessed once during pregnancy over a wide range (20 to 35 weeks).

## 5. Conclusions

Our preliminary data show that moderate-intensity PA during pregnancy seems to have a protective role against adverse maternal–fetal outcomes in women with GDM. This finding reinforces the importance of a physically active lifestyle during pregnancy in this population.

## Figures and Tables

**Table 1:** Characteristics of case and control participants.

Variables	n	Total	Case	Control	*p*-Value
n, %			24 (35.3)	44 (64.7)	
Age, years	68	32.06 ± 5.81	31.46 ± 4.79	32.39 ± 6.33	0.533
At least high school education, n (%)	68	28 (40.2)	8 (33.4)	20 (45.4)	0.251
Living with partner, n (%)	68	57 (83.8)	22 (91.7)	35 (79.5)	0.195
Family income over 1 minimum wage, n (%)	68	25 (36.8)	12 (50.0)	13 (29.5)	0.095
Gestational age, weeks 20–26 (second trimester) 27–35 (third trimester)	68	23 (33.8) 45 (66.2)	9 (37.5) 15 (62.5)	14 (31.8) 30 (68.2)	0.636
Parity, n (%) No gestation before One gestation before Two or more	68	16 (23.5) 16 (23.5) 36 (52.9)	4 (16.7) 6 (25.0) 14 (58.3)	12 (27.3) 10 (22.7) 22 (50.0)	0.613
Previous preterm birth, n (%)	67	7 (10.4)	3 (12.5)	4 (9.3)	0.695
Previous GDM, n (%)	68	5 (7.4)	2 (8.3)	3 (6.8)	1.000
Maternal family history of diabetes, n (%)	67	47 (70.1)	17 (70.8)	30 (69.8)	0.927
Pregestational BMI, kg/m^2^	68	28.50 (25.05–34.43)	29.20 (24.63–35.48)	28.50 (25.23–33.43)	0.734
Maternal weight gain (kg)	67	5.94 ± 7.18	6.04 ± 8.45	5.89 ± 6.53	0.934
Insulin therapy, n (%)	68	25 (36.8)	12 (50.0)	13 (29.5)	0.095
Hypertension, n (%)	68	25 (36.8)	12 (50.0)	13 (29.5)	0.095
Prenatal visits, n	64	9.38 ± 3.24	9.35 ± 3.79	9.39 ± 2.94	0.960
Mode of delivery Vaginal Cesarean	68	25 (36.8) 43 (63.2)	10 (41.7) 14 (58.3)	15 (34.1) 29 (65.9)	0.536

Abbreviations: GDM, gestational diabetes mellitus; BMI, body mass index. Continuous data are expressed as mean ± SD or median (25th to 75th percentiles). Categorical data are expressed as absolute (n) and relative (%) frequency.

**Table 2:** Physical activity and sedentary behavior measures during pregnancy of case and control participants.

Measures	Total		Case		Control		*p*-Value
	n		n		n		
**PA domains, MET-h/week**							
Leisure/exercise	21	0.00 (0.00–2.69)	5	0.00 (0.00–0.00)	16	0.00 (0.00–2.69)	0.160
Transportation	65	4.80 (1.50–7.53)	22	3.08 (1.50–7.15)	43	5.03 (1.73–8.66)	0.204
Household/domestic	67	52.37 (28.23–76.52)	24	46.22 (26.74–76.01)	43	55.61 (29.23–81.38)	0.492
Occupational	24	0.00 (0.00–48.00)	8	0.00 (0.00–66.72)	16	0.00 (0.00–48.00)	0.928
**PA intensities, MET-h/week**							
Light	68	67.75 (41.20–116.71)	24	58.35 (43.11–97.42)	44	71.38 (40.55–121.81)	0.457
Moderate	55	5.82 (1.92–19.88)	18	3.06 (0.45–10.30)	37	7.50 (3.63–22.87)	0.044 *
Vigorous	6	0.00 (0.00–0.00)	2	0.00 (0.00–0.00)	4	0.00 (0.00–0.00)	0.907
**Pedometer-measured PA, steps/day**	63	3.673 (2.809–4.792)	23	3.156 (2.703–4.727)	40	3.930 (2.933–4.989)	0.250
**Sedentary behavior, min/day**	68	416 (243–568)	24	418 (287–644)	44	405 (229–567)	0.626

Abbreviation: PA, physical activity. Continuous data are expressed as mean ± SD or median (25th to 75th percentiles). Categorical data are expressed as absolute (n) and relative (%) frequency. * Significant difference between groups (*p* < 0.05).

**Table 3:** Association between moderate-intensity physical activity during pregnancy and adverse maternal–fetal outcomes in women with gestational diabetes mellitus.

Moderate PA (MET-h/week)	Case	Control	Unadjusted		Adjusted	
	n	n	OR (95%CI)	*p*-Value	OR ^#^ (95%CI)	*p*-Value
Tertile 1 (0.00–3.60 MET-h/week)	12	11	Reference		Reference	
Tertile 2 (3.72–11.50 MET-h/week)	7	16	0.40 (0.12–1.34)	0.138	0.46 (0.12–1.80)	0.266
Tertile 3 (15.00–123.84 MET-h/week)	5	17	0.27 (0.07–0.98)	0.046 *	0.21 (0.05–0.91)	0.037 *

Abbreviations: PA, physical activity; CI, confidence interval. ^#^ Analysis adjusted for insulin therapy, hypertension, and intake of olive/vegetable oil and ultraprocessed food (Table S1). * Different from reference group (*p* < 0.05).

## Data Availability

The authors share the original database regarding outcomes of this study in the public repository for undetermined time, in order to independent verification of research results. The database can be found in: http://dx.doi.org/10.17632/5765ch5vn3.1.

## Supplementary Materials

The following are available online at https://www.mdpi.com/1660-4601/18/3/1114/s1, Table S1: Prevalence of appropriate dietary intake during pregnancy of case and control participants.
