# Supplementary material for: Movement Behavior during Pregnancy and Adverse Maternal–Fetal Outcomes in Women with Gestational Diabetes: A Pilot Case-Control Study
Source: Int J Environ Res Public Health. 2021 Jan 27;18(3):1114. doi: 10.3390/ijerph18031114 (PMC7908077; doi:10.3390/ijerph18031114)
Supplement: Supplementary file 1 [file ijerph-18-01114-s001.pdf]

Supplementary Table 1. Prevalence of appropriate dietary intake during pregnancy of case and control participants.

Sources, n (%)	n	Total	Case	Control	p-value
Vegetables	68	14 (20.6)	5 (20.8)	9 (20.5)	0.971
Fruit; natural juice	66	41 (62.1)	14 (60.9)	27 (62.8)	0.878
Nuts; chestnuts/oilseeds	68	6 (8.8)	2 (8.3)	4 (9.1)	1.000
Olive oil; vegetable oil	68	28 (41.2)	7 (29.2)	21 (47.7)	0.137
Whole grains	66	25 (37.9)	10 (45.5)	15 (34.1)	0.370
Sausages; processed meats	68	64 (94.1)	22 (91.7)	42 (95.5)	0.610
Milk; dairy products	68	29 (42.6)	11 (45.8)	18 (40.9)	0.695
Fish (Omega 3 sources)	68	20 (29.4)	9 (37.5)	11 (25.0)	0.280
Red meat with apparent fat	67	66 (98.5)	24 (100.0)	42 (97.7)	1.000
Soft drinks; artificial beverages	68	67 (98.5)	24 (100.0)	43 (97.7)	1.000
Ice cream; stuffed cookies	68	65 (95.6)	22 (91.7)	43 (97.7)	0.283
Fries or chips; fried snacks	67	67 (100.0)	24 (100.0)	43 (100.0)	-
Other ultra-processed foods	68	55 (80.9)	17 (70.8)	38 (86.4)	0.120
